# Off-Label Biliary Stents for Refractory Anastomotic Strictures and Leaks after Esophageal Atresia Repair in Infants

**DOI:** 10.1055/a-2879-7904

**Published:** 2026-06-11

**Authors:** Dmytro Rozov, Eisa Hag, Arcady Vachyan, Sobhi Abadi, Iyad Khamaysi

**Affiliations:** 1 Department of Pediatric Surgery 58878 Rambam Health Care CampusHaifaHaifa DistrictIsrael; 2 Department of Gastroenterology 58878 Rambam Health Care CampusHaifaHaifa DistrictIsrael; 3 Department of Radiology 58878 Rambam Health Care CampusHaifaHaifa DistrictIsrael; 4 58880 Technion Israel Institute of Technology, The Ruth and Bruce Rappaport Faculty of MedicineHaifaHaifa DistrictIsrael

**Keywords:** endoscopy upper GI tract, benign strictures, dilation, injection, stenting, pediatric endoscopy

## Abstract

Anastomotic strictures and leaks are frequent complications following the surgical repair of esophageal atresia, with a subset of patients developing refractory disease despite repeated dilations. This case series evaluates the preliminary feasibility and the outcomes of using off-label, fully covered, self-expanding, metal biliary stents in infants with refractory anastomotic strictures or leaks. Six infants treated at a tertiary referral center between 2021 and 2025 underwent endoscopic placement of fully covered self-expanding metal biliary stents. Technical success in stent placement and removal was achieved in all patients (100%). Clinical success was observed in 83.3% of patients; specifically, complete sealing of leaks and perforations was achieved in 100% of cases, while sustained luminal patency was observed in half of the isolated refractory strictures. One patient with a long-segment stricture required subsequent surgical revision. No major adverse events were identified during a median follow-up of 2.75 years. These preliminary findings suggest that biliary stents may represent a feasible rescue option for managing complex postoperative esophageal atresia complications in infants when specialized pediatric devices are unavailable.

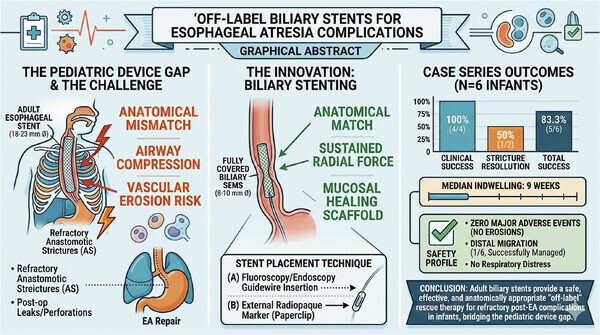

## Introduction

Anastomotic stricture (AS) is the most frequent complication following surgical correction of esophageal atresia (EA), with reported incidences ranging from 8% to 59% depending on the definition used. 1 2 Furthermore, anastomotic leaks represent a critical potential complication after surgery, often requiring aggressive management to prevent mediastinitis or chronic stricture formation. While 58–96% of cases respond to conventional dilation, approximately 30% develop refractory or recurrent strictures. 2 Predisposing factors include long-gap EA with anastomotic tension, postoperative leaks, gastroesophageal reflux disease (GERD), and suture material. 1

The use of standard adult esophageal stents in neonatal and infant populations is severely limited by a fundamental anatomic mismatch, as these devices are designed for a mature physiology that far exceeds the caliber and fragility of a child’s esophagus. When deployed in infants, the excessive diameter and high radial force of adult stents pose an unacceptable risk of transmural perforation and pressure-induced necrosis. Furthermore, due to the extreme proximity and pliability of surrounding structures, these oversized devices can cause extrinsic airway compression leading to respiratory distress or, more catastrophically, trigger vascular erosion into the aorta or other major thoracic vessels. 3 Adult biliary stents (8–10 mm diameter, 6–10 mm length) offer a smaller caliber suitable for pediatric esophageal dimensions and have been used off-label successfully in isolated cases. 4 5

This case series evaluates the safety and efficacy of fully covered self-expanding metal biliary stents (FC-SEMSs) in six infants with refractory AS or leaks post-EA repair.

## Methods

We conducted a retrospective review of six infants who underwent biliary stent placement for refractory AS or anastomotic leaks at our institution between 2021 and 2025 at a tertiary referral, university-affiliated hospital (Rambam Health Care Campus, Haifa).

All patients in our study had been diagnosed at birth to have EA and had subsequently developed strictures after anastomotic repair. We categorized primary indications for stent placement as refractory stricture, postoperative anastomotic leak (perforation), and postdilation esophageal leak (perforation). Refractory stricture was defined according to the joint ESPGHAN/NASPGHAN guidelines as the inability to achieve age-appropriate feeding due to persistent luminal narrowing despite five or more serial sessions of endoscopic dilation. 1 2

Patients were selected for biliary stent placement based on two primary clinical indications: (1) Refractory AS, defined as a persistent luminal narrowing that failed to resolve despite a minimum of five serial endoscopic balloon dilation sessions, resulting in an inability to achieve age-appropriate oral nutrition or (2) Anastomotic leak or iatrogenic perforation, where radiologic or endoscopic evidence of a mural defect was present and traditional conservative management was deemed insufficient or had failed to achieve closure. For this study, a “long-segment stricture” (as seen in Case 2) was defined as a fibrotic narrowing extending at least 20 mm in longitudinal length.

Data collection was performed via a review of medical records following institutional board committee approval. All procedures were performed in the operating room under general anesthesia to ensure patient safety, and the decision to proceed with stenting followed a comprehensive multidisciplinary discussion.

The procedure utilized the HANAROSTENT Biliary Lasso, an FC-SEMS manufactured by M.I. Tech Co., Ltd. (South Korea). These stents featured a delivery device diameter of 8Fr (2.66 mm) and were compatible with a 0.035-inch guidewire. Upper gastrointestinal endoscopy was performed using an Olympus EVIS EXERA III GIF-H190 video gastroscope (Olympus, Tokyo, Japan).

**Fig. 1:**
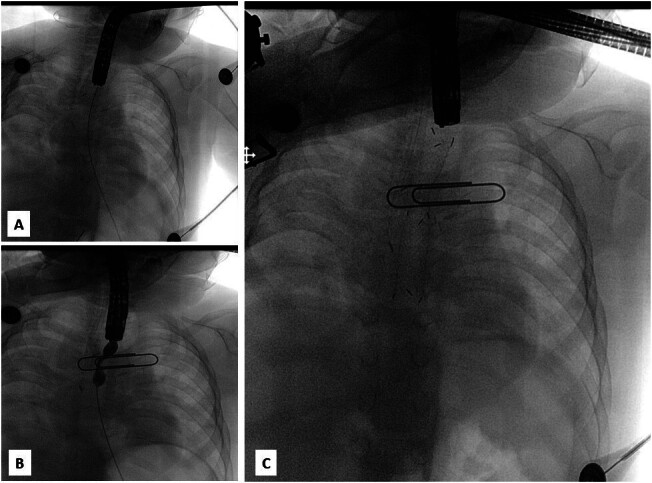
Stent placement technique. ( **A** ) Guidewire advanced across the lesion. ( **B** ) Delivery system positioned using an external radiopaque marker (paperclip) to identify the target. ( **C** ) Final deployment of the fully covered biliary stent (FC-SEMS) across the anastomosis.

The technical procedure began with a pre-stent assessment using a pediatric ultra-slim video gastroscope (GIF-XP190N, Olympus, Tokyo, Japan; outer diameter 5.4 mm, 2.2-mm working channel) to identify the lesion and measure the distance from the incisors. FC-SEMSs (8–10 mm diameter) were selected to extend 1–2 cm beyond the lesion margins. Under fluoroscopic guidance, a 0.035-inch guidewire was advanced into the stomach, the delivery system was positioned, and the stent was deployed across the anastomosis ( Fig. 1 ). Postprocedural care included aggressive acid suppression with proton pump inhibitors (PPIs) throughout the duration of stenting.

Proper stent placement was confirmed by endoscopy and fluoroscopy. After placement, serial chest radiographs were obtained every 24–48 h to evaluate for stent migration. All children were hospitalized for the duration of stent placement. Stent removal was accomplished by repeat endoscopy by using rat tooth forceps.

Adverse events documented were pain, retching, respiratory distress, stent migration, tissue ulceration, and granulation tissue.

Clinical success was defined according to the primary indication. For patients presenting with anastomotic leaks, success was defined as the complete radiologic resolution of the leak (confirmed by water-soluble contrast esophagram) and the avoidance of further surgical intervention. For patients with refractory strictures, success was defined as the resolution of obstructive symptoms—such as dysphagia or recurrent regurgitation—allowing for age-appropriate oral intake, alongside a stable or improving trend in growth percentiles during the follow-up period. Technical success was defined as the accurate endoscopic placement and successful deployment of the stent across the target lesion.

This study was approved by our Institutional Review Board (Rambam Health Care Campus Helsinki Review, 0092-13-RMB). Due to the lack of dedicated pediatric-sized esophageal stents, specific informed consent was explicitly obtained from the legal guardians of all infants prior to the procedure. This consent included a detailed explanation regarding the off-label use of FC-SEMSs, as well as a discussion of potential risks, benefits, and alternative treatment options.

## Results

### Patient Characteristics and Surgical History

The study cohort ( Tables 1 and 2 ) included six infants (3 males, 3 females) with a median weight of 9.65 kg (range 2.9–12.1 kg) and ages ranging from 2 weeks to 2 years. Underlying anatomy consisted of Type C EA in four cases and long-gap EA in two cases. Primary surgical interventions prior to stenting included thoracotomy ( *n* = 4) and the Foker procedure ( *n* = 2)—a technique utilizing internal traction sutures to stimulate esophageal growth and lengthening in long-gap EA cases prior to definitive anastomosis. Technical success in both stent placement and retrieval was achieved in 100% of cases.

**Table 1:** Patient characteristics and clinical indications.

Case	Sex	Age at procedure	Weight (kg)	EA type	Type of EA repair	Primary indication	Stricture length
1	Male	2 years	11.5	Type C	Thoracotomy	AS + Iatrogenic Perforation	N/A
2	Male	7 months	6.8	Long gap	Foker	Refractory AS	20 mm
3	Female	11 months	8.4	Long gap	Foker	AS + Iatrogenic Perforation	N/A
4	Male	2 years	10.9	Type C	Thoracotomy	Anastomotic Leak	N/A
5	Female	2 weeks	2.9	Type C	Thoracotomy	Anastomotic Leak	N/A
6	Female	2 years	12.1	Type C	Thoracotomy	Refractory AS	15 mm

AS: Anastomotic Stricture; EA: Esophageal Atresia

**Table 2:** Procedural specifications and clinical outcomes.

Case	Stent dimensions (Ø × L mm)	Indwelling time (weeks)	Adverse events	Clinical outcome	Follow-up (yrs)
1	10 x 60	19	None	Success; Leak sealed	4
2	10 x 60	14	None	Failure; Required Surgery	3
3	10 x 80	5	None	Success; Leak sealed	3.5
4	10 x 80	6	None	Success; Leak sealed	2.5
5	8 x 80	10	None	Success; Leak sealed	2
6	10 x 60	8	Distal Migration	Success; Patency maintained	0.5

### Clinical Outcomes

Clinical success was achieved in 83.3% (5/6) of the total cohort.

- **Leak and Perforation Management** : Fully covered FC-SEMSs were 100% successful (4/4) in sealing active anastomotic leaks and iatrogenic perforations.
- **Stricture Resolution** : Luminal patency was maintained in 50% (1/2) of isolated refractory strictures. The single clinical failure (Case 2) occurred in a patient with a 20-mm long-segment stricture following a Foker repair, who ultimately required surgical revision.
- **Outcomes by Indication: Leaks vs. Strictures:** Clinical outcomes varied significantly by indication. For patients with anastomotic leaks or perforations ( *n* = 4), clinical success was 100% (Cases 1, 3, 4, and 5); the biliary FC-SEMS provided an effective scaffold for complete tissue healing. Conversely, outcomes for refractory long-segment strictures ( *n* = 2) were mixed. While Case 6 was successful, Case 2—a 20 mm stricture following a Foker repair—failed to respond and required surgical revision. This suggests that while biliary FC-SEMSs are highly effective for sealing leaks, their ability to remodel complex, fibrotic long-segment strictures is more limited.

### Safety and Follow-up

The median indwelling time for the stents was 9 weeks (range 5–19 weeks). One instance of distal migration (Case 6) was successfully managed via endoscopic repositioning. No major adverse events, such as vascular erosion, tissue ulceration, or respiratory distress, were observed. Patients were followed for a median of 2.75 years (range 0.5–4 years), with all successful outcomes remaining stable.

## Discussion

This study demonstrates the technical feasibility of utilizing adult biliary stents as a rescue therapy for infants with refractory anastomotic complications following EA repair. Refractory ASs remain a formidable challenge in pediatric gastroenterology. While a universal definition is elusive, we adhered to the ESPGHAN/NASPGHAN guidelines, defining refractoriness as the failure to achieve age-appropriate feeding after five or more dilation sessions. 1 2 Furthermore, postoperative leaks are known precursors to chronic stricture formation, necessitating an internal scaffold to facilitate mucosal healing and prevent long-term morbidity.

Conventional management primarily relies on serial balloon dilation. While effective for simple strictures, fibrotic and refractory lesions often require frequent procedures with high recurrence rates. Pharmacologic adjuncts, such as intralesional steroids or mitomycin C, show inconsistent efficacy in infants and carry potential risks, including mucosal ulceration or theoretical concerns regarding malignancy. 5 6 In contrast, off-label biliary FC-SEMSs provide continuous radial force, acting as a dynamic dilator that facilitates tissue remodeling over several weeks—a distinct advantage over the transient stretch provided by balloons. For anastomotic leaks, stenting offers an immediate internal seal, allowing for early enteral nutrition and potentially avoiding aggressive surgical re-intervention. 4 7 8 9

In this series, adult biliary stents successfully addressed the “pediatric device gap.” Their 8–10 mm caliber is well suited for infant esophageal dimensions, providing sufficient luminal patency, while avoiding the high radial forces of adult esophageal stents, which risk transmural perforation or vascular erosion. Technical success was further supported by procedural flexibility; while a 0.035-inch guidewire remains our principal choice for stable delivery, these systems are compatible with 0.025-inch guidewires.

Regarding stent fixation, endoscopic clipping is a recognized technique to mitigate migration. However, we found that the radial force of the 10-mm biliary stents provided sufficient intrinsic anchorage in most cases. Distal migration occurred in only one patient (Case 4) and was successfully managed endoscopically. Given the relative caliber of the infant esophagus, routine anchoring with clips was not deemed necessary in this series, although it remains a viable adjunct for cases with recurrent migration.

The 100% success rate in the sealing of leaks and perforations (4/4) underscores the utility of FC-SEMS as an internal scaffold for tissue repair. However, these outcomes must be interpreted with caution. These results represent a preliminary experience in a highly selected, small cohort ($ *n* = 6$). The heterogeneity of the group—spanning neonates to toddlers and involving both acute leaks and chronic strictures—precludes broad generalizations. Our findings suggest these stents are a feasible rescue option, but their application requires careful patient selection and high-level endoscopic expertise.

The clinical failure observed in Case 2 (a 20-mm long-segment stricture) suggests that while biliary stents are effective for focal lesions, they may be insufficient for remodeling complex, long-segment pathologies. Safety in this series was prioritized through preprocedural cross-sectional imaging and aggressive PPI therapy to mitigate pressure necrosis and granulation tissue. Ultimately, these results emphasize the critical need for specialized, small-caliber pediatric esophageal hardware and support the initiation of larger, prospective multicenter studies to define the long-term safety and efficacy of this approach compared to standard pediatric interventions.

In conclusion, our initial experience suggests that the off-label use of fully covered biliary SEMSs may represent a feasible rescue option for refractory anastomotic complications following EA repair. While these stents provided a temporary internal scaffold that facilitated leak closure in this small cohort, their role in remodeling complex, long-segment strictures requires further investigation. Given the limited sample size ( *n* = 6) and the retrospective nature of this series, these observations should be considered preliminary and do not support routine clinical adoption at this stage. Prospective, multicenter studies are essential to rigorously evaluate the long-term safety profiles and comparative efficacy of this approach relative to standard pediatric interventions.
